# The association between living altitude and serum leptin concentrations in native women

**DOI:** 10.3389/fendo.2023.1107932

**Published:** 2023-02-22

**Authors:** Jiayu Cheng, Yingying Luo, Lihui Yang, Yufeng Li, Fang Zhang, Xiuying Zhang, Xianghai Zhou, Linong Ji

**Affiliations:** ^1^ Department of Endocrinology and Metabolism, Peking University People’s Hospital, Beijing, China; ^2^ Department of Endocrinology and Metabolism, Tibet Autonomous Region People’s Hospital, Lasah, China; ^3^ Department of Endocrinology and Metabolism, Capital Medical University Pinggu Teaching Hospital, Beijing, China

**Keywords:** serum leptin, altitude, native women, BMI, obesity

## Abstract

**Background:**

Lower diabetes prevalence and cardiovascular mortality have been observed in residents at a higher altitude. Leptin is associated with incident diabetes and adverse cardiovascular outcomes, and our aim was to investigate the association of living altitude with serum leptin concentrations.

**Methods:**

Two cross-sectional surveys were used in this study, including native populations living at Tibet (high altitude) and Beijing (low altitude). A propensity score was conducted for matching age and body mass index (BMI) between native women at high and low altitude. Pearson’s correlation analysis was performed to evaluate the correlation of leptin with other variables.

**Results:**

A total of 1414 native women were included in this study, including 594 at high altitude and 820 at low altitude. The serum leptin concentrations of native women living at high altitude were 13.74 ± 11.03 ng/ml, which was significantly lower than that of native women living at low altitude (20.90 ± 12.91 ng/ml). After matching age and BMI, women living at the high altitude still had lower serum leptin concentrations. After adjusting for the potential confounding factors, the correlation coefficient between Ln (leptin) and BMI of women at high altitude was significantly lower than that of women at low altitude (0.228 versus 0.559; *P <*0.0001). The serum leptin concentrations of each BMI subgroup (<18.5, 18.5 to <25, 25 to <30, ≥ 30 kg/m^2^) in women at high altitude were lower than that in women at low altitude.

**Conclusions:**

Serum leptin concentrations were significantly decreased in native women living at high altitude, and living altitude may alter the correlation of BMI and leptin. The findings of our study support that residents at high altitude have a protective effect with regards to improving cardiovascular and metabolic outcomes.

## Introduction

Obesity is a complex multifactorial disorder. Over the past decades, the prevalence of overweight and obesity has increased substantially, and one third of the world’s population is categorized into overweight or obese (1). In 2021, a nationally representative data reported that an estimated 85 million adults (48 million men and 37 million women) aged 18–69 years were obese in China (2). Obesity is a major risk factor for many diseases, including diabetes (3), cardiovascular diseases (4), renal diseases (5), and cancers (6).

Of note, overweight and obesity have divergent geographic prevalence and trends. Compared with the population living at low altitude, the counterparts living at high altitude has different metabolic characteristics, lower prevalence of obesity and diabetes (7, 8), and lower mortality of cardiovascular diseases (9). A previous study indicated that residents living at < 500 m had five times the risk of obesity than the residents living > 3000 m (10). Consistently, a cross-sectional study conducted at different altitudes reported that body mass index (BMI), waist circumference (WC) and waist-to-height ratio decreased with an increasing level of altitude (11). Therefore, the prevalence of obesity is inversely associated with altitude, which is independent of lifestyle and ethnicity (7). In addition, Americans living at high altitude is associated with lower adjusted risk of having diabetes compared to those living at low altitude (8). Furthermore, a longitudinal study showed that the mortality from coronary heart disease significantly decreased with increasing altitude (-22% per 1000 m), while adjusting for multiple risk factors (9). However, the mechanisms underlying the above interesting findings remain unknown.

Leptin is a peptide hormone mainly synthesized in white adipose tissue (12), and its circulating concentrations are typically proportional to the mass of body and fat (13). Leptin regulates food intake, body mass, glucose, lipid, and protein metabolism, and plays a vital role in cardiovascular disorders and proinflammatory immune responses (14, 15). A large prospective study indicated the association of high serum leptin concentrations with high risk of diabetes, and showed that serum leptin concentrations could predict incident diabetes (16). Besides, hyperleptinemia is positively correlated with adverse outcomes in cardiovascular diseases (15).

Few study have collected leptin values and other clinical data in the community at high altitude (17). In addition, it is unclear whether serum leptin concentrations and the association of leptin with other factors are the same for indigenous communities at high altitude as for other populations. Thus, our aim was to determine serum leptin concentrations and related factors of the native women at high altitude, and to find the supporting evidence of cardiovascular and metabolic health by comparing with the native women at low altitude.

## Methods

### Study population

We used two cross-sectional surveys, including native populations living at Tibet (>3500m above sea level; high altitude) and Beijing (0-100m above sea level; low altitude). In 2014, a study of endocrine disorders was carried out in community population of Tibet by two-stage cluster random sampling, and 1499 adults participated in the study. From September 2013 to July 2014, the Pinggu metabolic disease study was carried out in community population of Beijing by the same sampling, and 4002 adults aged 26-76 years old took part in the study. Details of the Pinggu metabolic disease study have been published in our previous publication (18). Pregnant women were not recruited in both studies. The exclusion criteria are as follows: (a) participants with diabetes history (n = 136 at high altitude; n = 386 at low altitude); (b) participants with newly diagnosed diabetes and prediabetes (fasting plasma glucose ≥ 6.1 mmol/L, and/or 2-h plasma glucose after a 75-g oral glucose tolerance test ≥ 7.8 mmol/L, and/or hemoglobin A1c ≥ 5.7%; n = 507 at high altitude; n = 1822 at low altitude); (c) participants with missing data on serum leptin concentrations (n = 18 at high altitude; n = 215 at low altitude). After excluding participants with the above criteria, 838 healthy individuals at high altitude and 1579 at low altitude were left. However, there was a large difference in the sample size of men between the two places (n = 244 at high altitude; n = 759 at low altitude), and there were few overweight and obese people at high altitude after BMI stratification, which may not be representative. Besides, there are substantial differences in fat mass and serum leptin concentrations between men and women. Thus, we finally enrolled 594 native women at high altitude and 820 native women at low altitude in the current analysis.

### The collection of clinical variables and biochemical parameters

Both the participants at high and low altitude underwent interviews with questionnaires, physical examinations and laboratory tests. We collect the data on demographic characteristics, height, weight, and blood pressure. BMI was calculated as weight divided by height in meters squared (kg/m^2^). After 10 min of rest, systolic blood pressure (SBP) and diastolic blood pressure (DBP) were measured.

Fasting blood samples for the measurement of the following biochemical parameters were collected. The biochemical parameters included fasting plasma glucose (FPG), and 2-hour post-prandial plasma glucose (2h-PPG) after a 75-g oral glucose tolerance test, hemoglobin A1c (HbA1c), triglycerides (TG), high-density lipoprotein cholesterol (HDL-C), low-density lipoprotein cholesterol (LDL-C), serum uric acid (UA) and serum leptin. HbA1c was measured by high-performance liquid chromatography (Adams A1c HA-8160; Arkray, Japan). Plasma glucose, TG, HDL-C, LDL-C, and serum UA were measured by an automated routine laboratory analyzer (UnicelDxC 800; Beckman Coulter, USA). Serum leptin concentrations were measured by a commercial ELISA kit (EMDMilipol, Billerica, MA, USA).

### Statistical analysis

Continuous variables are expressed as mean ± standard deviation (SD) or median (25th and 75th percentiles), and the differences between the two groups were compared by the t test or the Mann–Whitney test. Categorical variables are expressed as n (%), and the differences were compared by the chi-square test. To adjust the effect of age and BMI on serum leptin concentrations, we developed a propensity score matching for native women at high and low altitude. The propensity score through nearest neighbor matching was calculated using a multivariate logistic regression (19). Age and BMI were included in the logistic regression model. Due to the skewed distribution of serum leptin concentrations, its value was logarithmically transformed to Ln (leptin). Pearson’s correlation analysis was performed to evaluate the correlation between Ln (leptin) and other variables. Furthermore, serum leptin concentrations were calculated by BMI subgroups with the standard WHO criteria (<18.5, 18.5 to <25, 25 to <30, ≥ 30 kg/m^2^). The statistical significance level was set at P < 0.05. Statistical analysis was carried out using SPSS Statistics software (version 27.0).

## Results

### The characteristics of native women

As shown in **Table 1**, a total of 1414 native women were included in this study, including 594 at high altitude and 820 at low altitude. The mean age of women at high altitude was 35.6 ± 14.5 years and the BMI was 22.5 ± 3.8 kg/m^2^, while the mean age of women at low altitude was 44.0 ± 10.8 years, and the BMI was 24.6 ± 3.5 kg/m^2^. The women at high altitude had lower proportion of individuals with BMI ≥25 kg/m^2^ than those at low altitude. Compared with the women at low altitude, the women at high altitude had lower values of SBP, FPG, 2h-PPG, HbA1c, and LDL-C, but higher levels of TG, HDL-C, and serum UA (all *P <*0.001). The serum leptin concentrations of native women living at high altitude were 13.74 ± 11.03 ng/ml, which was significantly lower than that of native women living at low altitude (*P <*0.001). There was no significant difference of DBP between women at high and low altitude (*P >*0.05).

**Table 1:** The characteristics of native women living at high and low altitude.

	High altitude	Low altitude	*P* value
N	594	820	–
Age, y	35.6 ± 14.5	44.0 ± 10.8	<0.001
BMI, kg/m^2^	22.5 ± 3.8	24.6 ± 3.5	<0.001
BMI ≥25 kg/m^2^, n (%)	132 (22.2)	335 (40.9)	<0.001
SBP, mmHg	112.2 ± 15.9	121 ± 17	<0.001
DBP, mmHg	74.2 ± 11.1	74 ± 10	0.582
FPG, mmol/L	4.2 ± 0.5	5.2 ± 0.4	<0.001
2h-PPG, mmol/L	5.0 ± 1.1	6.1 ± 1.0	<0.001
HbA1c, %	5.1 ± 0.4	5.3 ± 0.2	<0.001
TG, mmol/L	0.94 (0.69, 1.41)	0.85 (0.56, 1.28)	<0.001
HDL-C, mmol/L	1.46 ± 0.45	1.26 ± 0.30	<0.001
LDL-C, mmol/L	2.07 ± 0.79	2.72 ± 0.72	<0.001
Serum UA, μmol/L	248 ± 60	234 ± 55	<0.001
Leptin, ng/ml	13.74 ± 11.03	20.90 ± 12.91	<0.001

BMI, Body mass index; SBP, Systolic blood pressure; DBP, Diastolic blood pressure; FPG, Fasting plasma glucose; 2-h PPG, 2-hour post-prandial plasma glucose; HbA1c, hemoglobin A1c; TG, Triglyceride; HDL-C, High-density lipoprotein-cholesterol; LDL-C, Low-density lipoprotein-cholesterol; UA, Uric acid.

To adjust the effect of age and BMI on serum leptin concentrations, an age and BMI-matched analysis was conducted between the native women at high and low altitude. **Table 2** shows that the age and BMI are comparable between the women at high and low altitude (*P >*0.05). However, compared with the women at low altitude, the women at high altitude had significantly lower serum leptin concentrations (13.50 ± 11.71 versus 16.01 ± 10.06 ng/ml).

**Table 2:** Serum leptin concentrations of native women living at high and low altitude after age- and BMI-matched.

	High altitude	Low altitude	*P* value
N	162	162	–
Age, y	40.3 ± 11.7	41.1 ± 11.0	0.535
BMI, kg/m^2^	23.2 ± 3.6	23.3 ± 3.2	0.872
Leptin, ng/ml	13.50 ± 11.71	16.01 ± 10.06	0.039

BMI, Body mass index.

### The correlation between leptin and different variables

**Figure 1:**
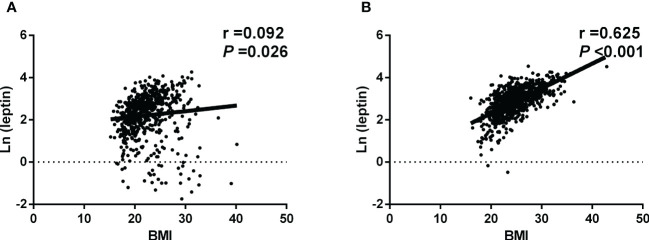
Scatter plot of BMI and Ln (leptin) **(A)**The native women living at high altitude. Pearson correlation analysis indicated that Ln (leptin) was mildly and positively correlated with BMI. **(B)** The native women living at low altitude. Pearson correlation analysis showed that Ln (leptin) was strongly and positively correlated with BMI.

**Figure 1** shows the scatter plot of BMI and Ln (leptin) of women living at high and low altitude. Pearson correlation analysis indicated that women at high altitude had a mild and positive correlation of Ln (leptin) with BMI (r = 0.092, *P* =0.026). The Ln (leptin) was strongly and positively correlated with BMI in women living at low altitude (r = 0.625, *P <*0.001).

In women at high altitude, the correlation coefficients indicated that the Ln (leptin) was positively correlated with HbA1c (r = 0.237, *P <*0.001) and TG (r = 0.122, *P* =0.003), while no significant correlations were found between Ln (leptin) with SBP, DBP, FPG, 2h-PPG, and UA (all *P >*0.05) (**Table 3**). In women at low altitude, the correlation coefficients suggested that the Ln (leptin) was positively correlated with DBP, FPG, 2h-PPG, HbA1c, TG, LDL-C, and UA (all *P <*0.01), while was negatively correlated with age (r = -0.120, *P* =0.001), and HDL-C (r = -0.207, *P <*0.001) (**Table 3**). After adjusting for the potential confounding factors, the correlation coefficient between Ln (leptin) and BMI of women at high altitude was significantly lower than that of women at low altitude (0.228 versus 0.559; *P <*0.0001) (**Table 3**).

**Table 3:** Correlation between Ln (leptin) and different variables of native women living at high and low altitude.

	High altitude		Low altitude	
	Coefficient (r)	*P* value	Coefficient (r)	*P* value
Without adjustment				
Age	-0.296	<0.001	-0.120	0.001
BMI	0.092	0.026	0.625	<0.001
SBP	-0.017	0.682	0.033	0.349
DBP	-0.010	0.800	0.168	<0.001
FPG	-0.058	0.157	0.143	<0.001
2h-PPG	-0.058	0.161	0.172	<0.001
HbA1c	0.237	<0.001	0.092	0.008
TG	0.122	0.003	0.237	<0.001
HDL-C	-0.204	<0.001	-0.207	<0.001
LDL-C	-0.121	0.003	0.125	<0.001
UA	-0.031	0.452	0.265	<0.001
After adjustment*				
BMI	0.228 (r1)	<0.001	0.559 (r2)	<0.001

P value for the difference between r1 and r2 was <0.0001.

*Adjusting for age, SBP, DBP, FPG, 2h-PPG, HbA1c, TG, HDL-C, LDL-C and UA.

BMI, Body mass index; SBP, Systolic blood pressure; DBP, Diastolic blood pressure; FPG, Fasting plasma glucose; 2-h PPG, 2-hour post-prandial plasma glucose; HbA1c, hemoglobin A1c; TG, Triglyceride; HDL-C, High-density lipoprotein-cholesterol; LDL-C, Low-density lipoprotein-cholesterol; UA, Uric acid.

### Serum leptin concentrations by BMI subgroups and menstruation

According to the BMI levels, the women at high and low altitude were categorized into four subgroups: BMI <18.5, 18.5 to <25, 25 to <30, ≥ 30 kg/m^2^. **Table 4** shows the mean ± SD, 2.5^th^, 25^th^, 50^th^, 75^th^, and 97.5^th^ percentile of serum leptin concentrations by BMI subgroups. The serum leptin concentrations of women with BMI of 18.5 to <25 kg/m^2^ at high altitude were 13.64 ± 9.65 ng/ml, while that of women with BMI of 18.5 to <25 kg/m^2^ at low altitude were 15.84 ± 9.04 ng/ml. Generally, the serum leptin concentrations of each BMI subgroup in women at high altitude were lower than that in women at low altitude. As shown in **Table 5**, both premenopausal women and menopausal/perimenopausal women at high altitude had lower serum leptin concentrations than those at low altitude.

**Table 4:** Serum leptin concentrations (ng/ml) of native women living at high and low altitude by BMI subgroups (kg/m^2^).

	Mean ± SD	2.5^th^, 97.5^th^	25^th^	50^th^	75^th^
High altitude					
All BMI (n=594)	13.74 ± 11.03	0.51, 42.41	5.29	11.59	18.71
<18.5 (n=71)	6.57 ± 5.51	0.65, 20.57	2.78	4.70	8.96
18.5 to <25 (n=391)	13.64 ± 9.65	0.81, 40.18	6.52	12.38	18.13
25 to <30 (n=108)	19.02 ± 13.11	0.37, 49.42	8.47	17.88	28.02
≥ 30 (n=24)	14.00 ± 19.51	0.24, -*	0.81	6.77	16.80
Low altitude					
All BMI (n=820)	20.90 ± 12.91	3.51, 54.86	11.95	17.95	26.72
<18.5 (n=17)	7.16 ± 5.91	1.41, -*	2.65	5.41	8.54
18.5 to <25 (n=468)	15.84 ± 9.04	3.30, 38.30	9.41	14.24	19.69
25 to <30 (n=272)	25.86 ± 11.19	9.05, 51.32	18.09	24.01	33.00
≥ 30 (n=63)	40.76 ± 16.58	14.46, 79.36	27.75	40.79	51.58

* Due to the number limitation of individuals, the 97.5^th^ of serum leptin concentrationss was not got.

BMI, Body mass index.

**Table 5:** Serum leptin concentrations (ng/ml) of native women living at high and low altitude by menstruation.

	Mean ± SD	2.5^th^, 97.5^th^	25^th^	50^th^	75^th^
High altitude					
premenopausal women (n=468)	14.61 ± 11.12	0.64, 44.43	6.27	12.71	19.81
Menopausal/perimenopausal women (n=95)	9.80 ± 10.43	0.39, 40.85	2.07	6.36	13.43
Low altitude					
premenopausal women (n=593)	21.40 ± 12.45	4.23, 54.86	12.53	18.72	27.30
Menopausal/perimenopausal women (n=227)	19.58 ± 13.97	2.47, 60.49	9.93	15.98	24.82

## Discussion

We found that native women living at high altitude had significantly lower serum leptin concentrations than the counterparts at low altitude, and the result was consistent even after matching age and BMI. In addition, the correlation between BMI and leptin of native women living at high altitude was weaker than that of women at low altitude. To the best of our knowledge, this is the first study with a large sample of representative population to compare serum leptin concentrations between local people at high and low altitude in China.

Some previous studies focused on the relationship between leptin and altitude. In a study of fifty-five healthy volunteer men, the data showed that there were no significant differences between plasma leptin concentrations in three populations of dwellers at different altitude (sea level, 3250 m, 4550 m) (17). Another study indicated that elevated plasma leptin concentrations were found after exposure to high altitude for 7 days in a group of 30 lowlanders (20). On the contrary, in a cross-sectional cohort of 889 subjects, the study reported an inverse correlation of serum leptin concentration with altitude (21). However, the above-mentioned studies have some limitations, including small sample size, unadjusted for BMI, and a relatively low altitude (200-1020 m) (17, 20, 21). After adjusting for age and BMI, this large sample study found lower serum leptin concentrations of native women at high altitude (>3500 m above sea level) compared to native women at low altitude (0-100 m above sea level). Besides, our study supports that women at high altitude may have favorable cardiovascular and metabolic profiles (7–9).

There are several potential mechanisms for the lower serum leptin concentrations at higher altitude. Hypoxia is an important feature of high altitude, which may be involved in the change of serum leptin concentrations. A study in rats showed that hypoxia exposure significantly reduced serum leptin concentrations, both in the exercise group and the non-exercise group (22). In addition, a study of healthy humans indicated that altitude-induced hypoxia suppressed plasma leptin concentrations (23). The increase in neural sympathetic activity at high altitude, partly induced by hypoxia, could inhibit leptin gene expression (24). Cold temperature also could play a role in the regulation of serum leptin concentrations. Recently, a prospective study reported that significant decrease of serum leptin concentrations were observed in healthy adults after short-term exposure to cold temperature (25). Of note, there are many confounders that have not been adequately addressed, which may have a combined effect on the relationship of leptin and altitude (26).

The data in our study reported that the correlation coefficient between BMI and leptin of native women living at high altitude was significantly lower than that of women at low altitude. We speculate that people at high altitude have less body fat with the same lean body mass, leading to the lower serum leptin concentrations (27). In concordance with our study, a previous study suggested that the high-altitude group had significantly higher HDL-C levels and lower BMI than the low-altitude group (28). Beside, compared with residents living below 500 m, clinically healthy residents living between 3000 and 4500 m had lower FPG (7). Lower prevalence of obesity and diabetes has been reported among people residing at high altitudes (29). Animal study showed that blockade of leptin signaling could decrease blood pressure (30), supporting that low serum leptin concentrations contribute to the favorable cardiometabolic outcomes.

Our study has several limitations. Firstly, the participants living at high altitude were Tibetans, and the participants living at low altitude were Han Chinese. the ethnic variation in our study may contribute to the difference of serum leptin concentrations. However, a previous study indicated that ethnicity may not explain the favorable metabolic profiles of residents at higher altitudes (8). Beside, Tibetans have lived at Tibet for generations, while Han Chinese have lived at low altitude for generations, and we speculate that they have genetic homogeneity. Secondly, our study did not have the information of dietary pattern and physical exercise. Of note, the dietary habits and physical exercise could lead to the change of BMI levels, but we adjusted for age and BMI when we analyzed the leptin concentrations. Thirdly, we did not collect the data on fat distribution, visceral fat and subcutaneous fat, which may help us understand the relationship between BMI and leptin. Fourthly, our study analyzed the data of native women. In a study of women and men, the authors reported an inverse correlation of leptin and altitude (21), but it needs more studies to verify.

In conclusion, our study found that serum leptin concentrations were significantly decreased in native women living at a higher altitude, and living altitude may alter the correlation of BMI and leptin. The findings of our study support that residents at high altitude have a protective effect with regards to improving cardiovascular and metabolic outcomes. Future studies are required to verify the findings in our study and clarify the potential explanations between leptin, altitude and cardiometabolic outcomes.

## Data availability statement

The data analyzed in this study is subject to the following licenses/restrictions: The original contributions presented in the study are included in the article. Further inquiries can be directed to the corresponding author. Requests to access these datasets should be directed to XiaZ, xianghai_zhou@bjmu.edu.cn.

## Ethics statement

The studies involving human participants were reviewed and approved by the medical ethics committees of Tibet Autonomous Region People’s Hospital and Peking University Health Science Center. The patients/participants provided their written informed consent to participate in this study.

## Author contributions

XiaZ and LJ contributed to the study concept and design. LY, YuL, FZ, and XiuZ contributed to the acquisition of data. JC performed the statistical analysis. JC and YiL were involved in interpretation of the data. All authors contributed to drafting, modifying and approving the manuscript, and take responsibility for accuracy and integrity of the manuscript. All authors contributed to the article and approved the submitted version.
